# A novel accelerometry-based algorithm for the detection of step durations over short episodes of gait in healthy elderly

**DOI:** 10.1186/s12984-016-0145-6

**Published:** 2016-04-19

**Authors:** M. Encarna Micó-Amigo, Idsart Kingma, Erik Ainsworth, Stefan Walgaard, Martijn Niessen, Rob C. van Lummel, Jaap H. van Dieën

**Affiliations:** MOVE Research Institute Amsterdam, Department of Human Movement Sciences, Vrije Universiteit Amsterdam, Amsterdam, The Netherlands; McRoberts B. V., Raamweg 43, 2596 HN The Hague, The Netherlands

**Keywords:** Analysis of short episodes of gait, Body-fixed-sensors, Accelerometers, Low-back accelerometry, Heel accelerometry, Step duration detection, Healthy elderly

## Abstract

**Background:**

The assessment of short episodes of gait is clinically relevant and easily implemented, especially given limited space and time requirements. BFS (body-fixed-sensors) are small, lightweight and easy to wear sensors, which allow the assessment of gait at relative low cost and with low interference. Thus, the assessment with BFS of short episodes of gait, extracted from dailylife physical activity or measured in a standardised and supervised setting, may add value in the study of gait quality of the elderly. The aim of this study was to evaluate the accuracy of a novel algorithm based on acceleration signals recorded at different human locations (lower back and heels) for the detection of step durations over short episodes of gait in healthy elderly subjects.

**Methods:**

Twenty healthy elderly subjects (73.7 ± 7.9 years old) walked twice a distance of 5 m, wearing a BFS on the lower back, and on the outside of each heel. Moreover, an optoelectronic three-dimensional (3D) motion tracking system was used to detect step durations. A novel algorithm is presented for the detection of step durations from low-back and heel acceleration signals separately. The accuracy of the algorithm was assessed by comparing absolute differences in step duration between the three methods: step detection from the optoelectronic 3D motion tracking system, step detection from the application of the novel algorithm to low-back accelerations, and step detection from the application of the novel algorithm to heel accelerations.

**Results:**

The proposed algorithm successfully detected all the steps, without false positives and without false negatives. Absolute average differences in step duration within trials and across subjects were calculated for each comparison, between low-back accelerations and the optoelectronic system were on average 22.4 ± 7.6 ms (4.0 ± 1.3 % of average step duration), between heel accelerations and the optoelectronic system were on average 20.7 ± 11.8 ms (3.7 ± 1.9 %), and between low-back accelerations and heel accelerations were on average 27.8 ± 15.1 ms (4.9 ± 2.5 % of average step duration).

**Conclusions:**

This study showed that the presented novel algorithm detects step durations over short episodes of gait in healthy elderly subjects with acceptable accuracy from low-back and heel accelerations, which provides opportunities to extract a range of gait parameters from short episodes of gait.

## Background

Gait laboratory equipment usually consists of a combination of force plates, pressure-sensitive foot switches and an optical motion capture system. The assessment of human gait with such systems provides objective and quantitative measurements. Nonetheless, these systems also present several important constraints, such as the high cost of the equipment, the limited number of steps to assess [1] (over ground walking) or an induced gait speed (treadmill) [2], and the limited ecological validity, i.e., the natural environment where the physical activity of the subject normally takes place is not replicated, which may affect the outcomes [3, 4].

As an alternative to laboratory equipment, body-fixed-sensors (BFS) are small, lightweight, easily wearable and highly transportable. In addition, limited interference with activity is expected and low power is required [1], which permits an ambulatory use of these sensors for long-duration measurements (up to two consecutive weeks [5]) at relative low cost, especially when using a single sensor.

BFS might also be useful in simple supervised assessment protocols, for example to analyse short episodes of gait, which can easily be included in clinical assessment, especially given its limited space and time requirements, and given that physical limitations in some patients might be an impediment to perform longer episodes [6, 7]. Moreover, older adults select gait strategies with different spatio-temporal parameters for different distances, therefore, the assessment of short episodes of gait has potential to provide clinical information that differs from information based on the assessment of long episodes of gait [8]. The relevance of clinical assessment of short episodes of gait with BFS has been supported in several studies [8–10]. For instance, a study based on quantitative assessment of the 8 m gait test with two gyroscopes placed at the level of L1-L3 supported the ability to differentiate between fall-prone and healthy elderly subjects [9]. Furthermore, it has been proven that data from a single BFS worn during the assessment of the Timed Up and Go test (rising from a chair, walking 3 m, turning, walking back and sitting down on the chair) might distinguish between different clinical subtypes of Parkinson’s Disease [10]. In addition, the assessment of short episodes of gait with BFS is well suited to study the initiation of gait [11], which has been shown to be clinically relevant in diseases such as Parkinson’s Disease [12, 13].

A relevant challenge in the assessment of spatio-temporal and stability gait parameters using BFS is the accurate and reliable detection of steps [14, 15], which is related to the placement of the sensor [16, 17]. Particularly, the heels seem to be an adequate location for the placement of accelerometers to detect heel-strikes due to the magnitude of the acquired acceleration signals [18] and the proximity to the location where the ground reaction force impacts [19]. From this perspective, the placement on the lower back might not be the most suitable location for step detection. However, low-back accelerometry does permit the detection of gait events [19–28] and the extraction of spatio-temporal gait parameters [16, 20, 25, 26, 28, 29]. In addition, due to its proximity to the centre of mass, the acquired low-back acceleration represents the overall human motion pattern [17]. Additionally, trunk accelerometry permits the assessment of fall risk [14, 30], trunk stability [31, 32] and balance control [33, 34], which combined with step detection might be clinically relevant. However, a step-based analysis is only valid if step cycles are detected with sufficient accuracy [20].

Previous studies have identified gait events, using a single BFS at the lumbar level [19–23, 25, 28], or two BFS on the heels [18, 35]. However, incorrect identification of events and/or miss-detection of events were found when applying peak-based methods [1, 18–23, 35, 36]. These methods depend on specific properties of acceleration signals at specific instants of time [19]. Therefore, they might be sensitive to fluctuations between steps (especially in short episodes of gait) and between patients (due to different walking patterns or different sensor alignment), which leads to erroneous detection of events. On the other hand, wavelet signals processing techniques, as previously proposed [19, 23, 25, 26], require a relative large number of complete periods [37], which are not available from short episodes of gait. Moreover, the use of these techniques does not always guarantee the detection of all gait events [19, 25, 26].

In this study, we present a novel algorithm for detection of step durations over short episodes of gait in healthy elderly subjects. The proposed algorithm was separately applied to acceleration signals recorded at different locations: lower back and heels. The method aims to identify all periodic intervals from acceleration signals which resemble in shape and magnitude to a predefined template. This template is individualized and defined as a combination of all cycles within each episode of gait. Thus, the success of this method in step duration detection does not depend on special features of acceleration signals at specific events, but on the shape and magnitude of acceleration signals along periods. Furthermore, based on an individualized and averaged template-match principle, low sensitivity to subjects fluctuations is expected, allowing proper detection of all cycles from different periodic acceleration signals of short duration.

In addition to the accelerometers, an optoelectronic 3D motion tracking system was used to detect step durations. Optoelectronic motion capture systems, while not the gold standard, are often used given their highly accurate 3D measurements of position and may provide an additional basis for accurate step detection [18, 38, 39]. The accuracy of the algorithm was evaluated by comparing absolute differences in step duration between the estimates obtained from the three methods: step detection from the optoelectronic 3D motion tracking system, step detection from the application of the algorithm to low-back accelerations, and step detection from the application of the algorithm to heel accelerations.

## Methods

### Subjects

This cross-sectional study was performed with a group of 20 healthy older adults (9 female and 11 male, average age 73.7 ± 7.9 years, average height 173.3 ± 8.2 cm, average leg length 105.3 ± 5.2 cm, average weight 77.7 ± 13.1 kg). The subjects, who were recruited from an ongoing cohort study concerning fall risk assessment in older adults (FARAO), were community dwelling elderly adults. They were recruited in Amsterdam (the Netherlands) and its surroundings.

Once the protocol was approved by the local Ethics committee (METc VUmc: 2010/290), the subjects were selected from the FARAO cohort according to the following inclusion criteria: (a) age between 65 and 99 years; (b) mini mental state examination score of at least 19 points out of 30; (c) able to walk 20 m without any walking aid and without any cardiovascular or respiratory symptoms. Prior to the measurements in the laboratory at the Department of Human Movement Sciences (Vrije Universiteit Amsterdam), the selected subjects provided informed written consent for the participation in the study and for the publication of individual, anonymized data.

### Protocol

The subjects, wearing their own shoes, walked a 5 m long track demarcated by two lines on the floor. Two paper templates of two adult-sized footprints were attached to the floor indicating the end position of the track. Two trials were performed at preferred speed.

At the beginning of each trial the subjects stood behind the start line with the toes placed on the line. After a verbal countdown the subjects started to walk the distance marked by the lines on the floor. The trial ended when the subjects reached the end line, with their shoes placed on the footprint templates. Afterwards the subject stood still for 3 s before returning to the starting position and repeating the trial.

### Instrumentation and data acquisition

A BFS (DynaPort Hybrid, McRoberts; 87 mm x 45 mm x 14 mm, 65 g) was inserted in an elastic belt, placed around the waist in such a way that the sensor was positioned at the level of the lowest lumbar vertebra (L5). This location is well accepted by older adults [40] and it is close to the centre of mass of the whole human body [17, 41]. The system includes a triaxial accelerometer and a triaxial gyroscope storing data at a sampling rate of 100 samples/s. The accelerometer has a resolution of 1 mg and it is a DC type sensor, therefore it is also sensitive to gravity. During the standing phase, the sensor inside the belt was approximately parallel to the coronal plane.

The second system was composed of two BFS (DynaPort MiniMod McRoberts; 82 mm x 50 mm x 9 mm, 43 g), attached to the lateral sides of both heels. These BFS included a DC type triaxial accelerometer with a sample rate of 100 samples/s and a resolution of 1 mg. Each sensor was attached with tape to the lateral side of the shoe (close to the heel).

**Fig. 1:**
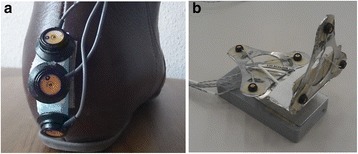
**a** Location of the LED markers of the Optotrak system on one of the heels. **b** Cluster of markers placed on a lightweight plate and attached to the surface of the DynaPort Hybrid, McRoberts

The third system was an optoelectronic three-dimensional (3D) motion tracking system, 3020 Optotrak, Northern Digital Inc., Waterloo, Canada, consisting of three camera arrays for the recording of the 3D position (error < 0.05 mm) of LED markers at a sample rate of 200 samples/s. The LED markers were positioned as follows: three single markers on the back side of both shoes (Fig. 1a) and two clusters of three markers on the lower back (L5), placed on a lightweight plate which was attached to the surface of the DynaPort Hybrid (Fig. 1b).

Data acquisition was synchronized for all the collected signals using an impulse, which was transferred to each of the systems. In addition, a video camera was used to record the measurements.

### Step detection from low-back (L5) accelerometry

**Fig. 2:**
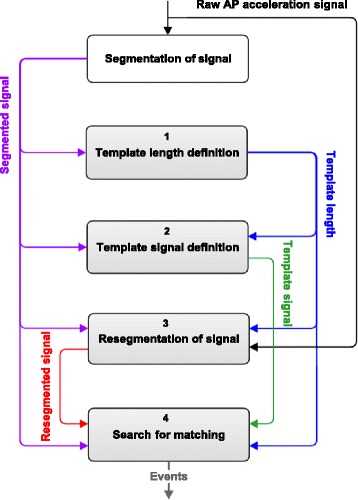
Flowchart that represents the operations executed by the algorithm

A novel algorithm was developed for the automatic detection of step durations from acceleration signals. The algorithm is based on the acceleration in the anterior-posterior (AP) direction. Firstly, it defines a template, which represents a typical pattern of a step cycle acceleration, and subsequently it searches for the periods of maximal match between the signal and the template. The raw acceleration signal obtained from the accelerometers was manually segmented from some samples before the first heel-strike of the gait episode to some samples before the foremost foot reached the end line. The result is denoted as “Segmented signal”. Based on low-back accelerometry and following the flowchart (Fig. 2), the algorithm executes the subsequent operations:

1. Template length definition
  In this block the length of the template signal was defined using the “Segmented signal” as an input and following these steps:
  1.1. Obtain the unbiased autocorrelation signal of the input with the function < xcov, unbiased > from Matlab Signal Processing Toolbox 7.11.0 [29].
  1.2. Extract the dominant frequency from the unbiased autocorrelation signal (positive lags).
  1.3. Calculate the inverse of the dominant frequency and multiply it by the sampling frequency. The Template Length (TL) is defined as the resulting number of samples.
2. Template signal definition
  In this block the “Template signal” is defined, using TL and the “Segmented signal” as inputs.
  2.1. Define a low limit on the “Segmented signal” after 115 % TL samples from the start, and a high limit 115 % TL samples before the end.
  2.2. Find peaks in the “Segmented signal” between the low and high limits, which are at least 40 % TL samples from each other.
  2.3. Define sections of the input signal around each peak, starting 15 % TL samples before the instant at which the peak is found (with the aim to include the slope preceding the peak) and ending 100 % TL samples after the instant at which the peak is found.
  2.4. Obtain new signals from the application of the dynamic-time-warping technique (DTW) [42] on each of the sections and their consecutive ones. This technique optimally aligns the sections, combining them in an average signal.
  2.5. Repeat step 2.4 until a single new signal, named “Template signal”, is obtained. The length of this signal is TL samples. This signal is the average of all the sections defined in step 2.3 (Fig. 3).

**Fig. 3:**
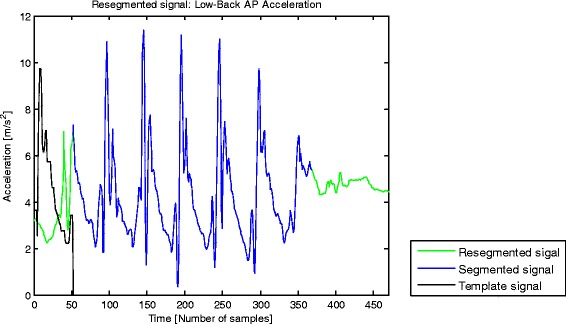
“Resegmented signal”, “Segmented signal” and “Template signal” with a sampling rate of 100 samples/s. Typical example of an AP acceleration signal collected at the lower back and segmented from shortly before the first heel-strike of the gait episode until shortly before the foremost foot reached the end of the trial (“Segmented signal”, blue). New segmentation of the raw AP acceleration signal, which contains three sections with a number of samples equivalent to the template length, one section prior to the start marker of the initial segmentation, and two sections after the end marker of the initial segmentation (“Resegmented signal”, green). “Template signal” (black) is the average of all the sections defined in step 2.3
3. Resegmentation of signal
  In this block a new segmentation of the raw acceleration signal is performed using TL, the raw acceleration signal, and the start and end sample number of the “Segmented signal” as inputs.
  3.1. Extend the segmentation of the “Segmented signal” to the left with TL samples, and to the right with twice TL samples from the original raw acceleration signal. The resulting signal is denoted as “Resegmented signal” (Fig. 3), the output of this block.
4. Search for match between “Template signal” and “Resegmented signal”
  In this block the match between the “Template signal” and the “Resegmented signal” is found. The aim is to extract the periods from the “Resegmented signal” in which the acceleration resembles the template in magnitude and shape. This permits to evaluate the periodicity of steps. The inputs of this block are template length (TL), the “Template signal”, the ¨Segmented signal” and the “Resegmented signal”.
  4.1. Calculate a signal based on the standard deviation of the difference in amplitude between the “Template signal” and a sliding window (with TL samples) through the “Resegmented signal”.
  4.2. Normalize the resulting signal. This signal, denoted as “SD Difference signal”, has local minima at the start of the intervals along which the “Resegmented signal” and the “Template signal” have the best match, and therefore are more similar in shape and amplitude (Fig. 4a).

**Fig. 4:**
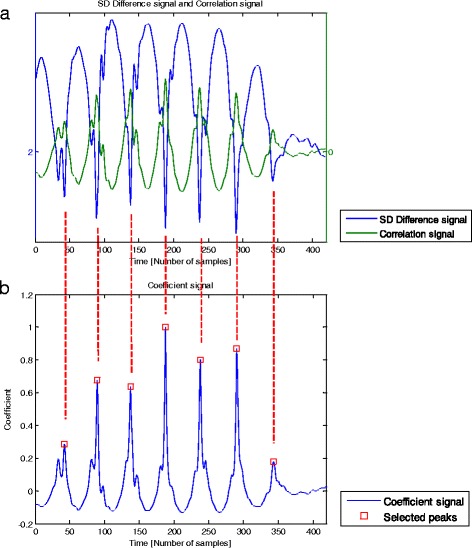
**a** Typical example of a “SD Difference signal” (blue) and a “Correlation signal” (green) with a sampling rate of 100 samples/s. The “SD Difference signal” was obtained from the standard deviation of the difference in amplitude between the “Template signal” and a sliding window (with a number of samples equivalent to the template length) through the “Resegmented signal”. This signal has lower values at the start of the intervals along which the “Resegmented signal” and the “Template signal” are more similar in shape and amplitude. “Correlation signal” was obtained from the calculation of correlation coefficients between the “Template signal” and a sliding window through the “Resegmented signal”, being multiplied by the ratio of ranges of the “Resegmented signal” and the “Template signal”. The resulting signal has higher values at the start of the intervals along which the “Resegmented signal” and the “Template signal” are more similar in shape and amplitude. **b** “Coefficient signal” (blue) and “Selected peaks” (red squares) with a sampling rate of 100 samples/s. The normalized ratio signal between the “Correlation signal” and the “SD Difference signal” permitted to obtain the “Coefficient signal”. “Selected peaks” are the peaks from the “Coefficient signal” which are found within the dimension of the “Segmented signal” and are located at a distance of at least a 60 % TL (template length) samples from each other
  4.3. Calculate a signal based on the calculation of correlation coefficients (using the function < corrcoef > from Matlab Signal Processing Toolbox 7.11.0) between the “Template signal” and a sliding window through the “Resegmented signal”.
  4.4. Multiply the resulting signal by the ratio of ranges of the “Resegmented signal” and the “Template signal” and normalize the result. This signal, denoted as “Correlation signal”, has local maxima at the start of the intervals along which the “Resegmented signal” and the “Template signal” have the best match, and therefore are more similar in shape and amplitude (Fig. 4a). Note that both the “SD Difference signal” and the “Correlation signal” are TL samples shorter than the “Resegmented signal”.
  4.5. Calculate the ratio between the “Correlation signal” and the “SD Difference signal” to obtain a new signal, denoted as “Coefficient signal” (Fig. 4b).
  4.6. Select peaks in the “Coefficient signal” which are found within the dimension of the “Segmented signal” and are located at least a 60 % TL samples distant to each other.
  4.7. Shift forwards by 15 % TL samples the instants at which the peaks were selected, in order to define the instants that approximate heel-strike events in time. The intervals defined between the shifted peaks, named “Events”, allow step durations to be calculated (Fig. 5a).

**Fig. 5:**
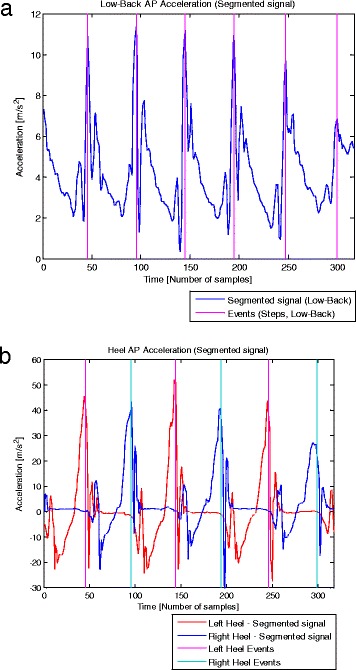
**a**. Low-Back acceleration signal (with a sampling rate of 100 samples/s) and detected events. Typical example of a segmented AP acceleration signal collected at the lower back (blue), and the “Events” (magenta) detected by the algorithm applied to low-back accelerometry. These “Events” were obtained from the shift by a 15 % TL samples of the instants at which the peaks were found. The intervals between “Events” permitted to calculate step durations. **b**. Heel acceleration signals (with a sampling rate of 100 samples/s) and detected events. Typical example of segmented AP acceleration signals collected at the heels, left heel (red) and right heel (blue), and the respective “Events” detected by the algorithm. These “Events” were obtained from the shift by a 5 % TL samples of the instants at which the peaks were found. The intervals between “Events” calculated for each of the heel acceleration signal correspond to stride durations, and the combination of the events detected from both heel accelerometry permitted to obtain step durations

### Step detection from heel accelerometry

Acceleration signals of both heels were synchronized with low-back acceleration signals and segmented at the same point. The algorithm applied to the heel accelerations was similar to that of low-back accelerations. However, since heel accelerations have a periodicity in strides [18] instead of steps, the template represents a typical pattern of a stride cycle acceleration and it is differently defined. The following steps of the algorithm are different for heel accelerometry.

1.3. The template length (TL) is defined as the resulting number of samples between the first two peaks of the low-pass filtered (cut-off frequency equivalent to the double dominant frequency) normalized unbiased autocorrelation signal which overcome a threshold of 0.5.
2.3. Define sections of the input signal around each peak, starting 5 % TL samples before the instant at which the peak is found (instead of 15 %, as the slope of the peaks is more steep and TL is about twice long for heel accelerations compared to low-back accelerations) and ending 100 % TL samples after the instant at which the peak is found.

In the case of heel accelerometry, the intervals defined between the shifted peaks (5 % TL samples), named “Events”, permit stride durations to be calculated. Thus, left and right stride durations are detected from their respective acceleration signals, and these are combined to obtain step durations (Fig. 5b).

### Step detection from Optotrak data

Heel and low-back acceleration signals were sampled at 100 samples/s, whereas Optotrak signals were sampled at 200 samples/s. In order to obtain the same sample rate between the three systems, Optotrak signals were resampled to 100 samples/. In addition, Optotrak signals were synchronized with acceleration signals and segmented at the same point. The detection of heel-strike events from the Optotrak system was influenced by the visibility of the LED markers. These markers were not continuously visible, therefore, the visibility of Optotrak signals was evaluated within intervals of 40 samples around the instants of the *“Events”* detected from heel accelerations. The visibility of Optotrak signals within each interval was evaluated for all LED markers. From the markers placed on the same area (right heel, left heel and lower back), the one which had the largest number of complete visible intervals in the whole gait episode was selected. Heel-strike events were estimated as the instants when the distance in anterior-posterior direction was maximal between the selected heel and low-back markers [38].

### Comparison between different systems

Step durations were calculated as the intervals between the *“Events”* obtained with each method. The comparison was based on the calculation of absolute differences (in milliseconds) in step duration between methods for every step. However, in the case of Optotrak, 8.9 % of the heel-strike events were missed due to the lack of continuous visibility of the selected LED marker along the defined intervals. As a consequence, the respective heel-strike events were not included in the calculation of step durations, and instead, a stride duration was calculated and compared.

Absolute differences in step duration and/or stride duration (in the cases of missed heel-strike events) between each pair of methods were calculated and averaged across all the steps of both trials, for all the subjects. Shapiro-Wilk tests were applied to check the normal distribution of these differences. In case of a normal distribution, differences were compared with a paired *t*-test. In case of a non-normal distribution, a Wilcoxon Signed Rank test was used. Furthermore, interclass correlation (ICC) were used to assess correspondence between methods. Significance level was set at α = 0.05.

## Results

**Fig. 6:**
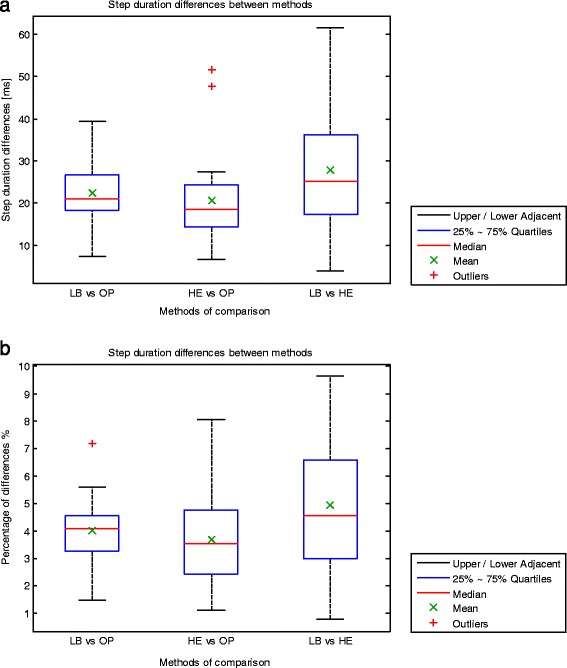
Average absolute differences in step duration within trials and for each subject are presented for each pair of compared methods with a boxplot (**a**). Percentage of average absolute differences in step duration over the average step duration calculated with both systems within trials and for each subject are presented for each pair of compared methods with a boxplot (**b**). *LB = low-back accelerometry, HE = heel accelerometry, OP = Optotrak. The boxplot includes minimum, first quartile (q1, 25 %), median, third quartile (q3, 75 %), maximum and outlier values

A Shapiro-Wilk’s test showed that absolute differences between methods did not deviate from normal distribution for absolute differences in step duration between low-back accelerometry and Optotrak (*p* = 0.97) and between low-back accelerometry and heel accelerometry (*p* = 0.50). However, when comparing estimates from heel accelerometry versus Optotrak, absolute differences were not normally distributed (*p* = 0.01), with the presence of two outliers (Fig. 6).

The number of steps performed by the subjects were counted from video recordings, which confirmed that the algorithm applied to both low-back and heel accelerations detected an equal number of steps, without false positives and without false negatives.

Step duration estimates calculated from low-back accelerations were compared with estimates from Optotrak data. Absolute differences within episodes and across subjects ranged between 7.5 and 39.2 ms. The ICC of step durations over the two methods was 0.91. The average absolute difference was 22.4 ± 7.6 ms, which is 4.0 ± 1.3 % of the average step duration calculated with both systems (Fig. 6). Estimates calculated from heel accelerations were compared with estimates from Optotrak data. Absolute differences within episodes and across subjects ranged between 6.7 and 51.5 ms. The ICC of step durations over the two methods was 0.91. The average absolute difference was 20.7 ± 11.8 ms, which is 3.7 ± 1.9 % of the average step duration measured with both systems (Fig. 6). Estimates calculated from low-back accelerations were compared with estimates from heel accelerations. Absolute differences within episodes and across subjects ranged between 4.0 and 61.4 ms. The ICC of step durations over the two methods was 0.88. The average absolute difference was 27.8 ± 15.1 ms, which is 4.9 ± 2.5 % of the average step duration measured with both systems (Fig. 6).

The ICC of step durations across the three methods was 0.90. Differences between estimates from heel accelerations and Optotrak, which yielded the lowest average value, were not statistically significant different from differences between estimates from low-back accelerations and Optotrak (*p* = 0.46), although they were statistically significant different from differences between estimates from heel and low-back accelerations (*p* = 0.02). Differences between estimates from low-back and heel accelerations yielded the highest average absolute value, and these were not statistically significant different from differences between estimates from low-back accelerations and Optotrak (*p* = 0.16).

## Discussion

In this study, we analysed the accuracy of a new algorithm for the detection of step durations in healthy elderly subjects during short episodes of gait. The proposed algorithm was applied separately on low-back and heel accelerations, and its accuracy was evaluated by comparing estimates from three methods: step detection from an optoelectronic 3D motion tracking system (Optotrak), step detection from the application of the algorithm on low-back accelerations, and step detection from the application of the algorithm on heel accelerations.

The lowest differences were obtained when comparing estimates from heel accelerations and Optotrak (3.7 ± 1.9 %). These differences were significantly lower than differences between estimates from heel accelerations and low-back accelerations (4.9 ± 2.5 %). However, these differences were non-significantly lower than differences between estimates from low-back accelerations and Optotrak (4.0 ± 1.3 %). Part of the differences between methods may be due to the measurement at different parts of the body, the misalignment between left/right sensors and markers, and the use of different type of signals (position versus acceleration). As a result, the three methods may detect different gait events. Nonetheless, as long as the periodicity of the detected events is the same, the duration of steps should be comparable between methods.

Gait cycles are within inertial data reproduced as repeating patterns [16]. Based on this principle, the proposed algorithm searches for the periods of maximal match between signals and an individualized and averaged template, which represents a typical acceleration pattern of a step cycle (in case of low-back acceleration) or a stride cycle (in case of heel acceleration). Likewise, there are potential applications of the proposed algorithm to other periodic signals, considering the proper definition of a template that will represent the typical pattern of a period of interest.

The magnitude and shape of input signals is related to the location of the sensor [16, 17]. In this regard, the heels seem to be the most adequate location of the accelerometers to detect heel-strikes, since the peaks are steeper. However, the placement of accelerometers on the heels has some disadvantages in clinical practice as two sensors are required for the calculation of step durations, with both sensors being attached to the shoes. Self-attachment of the sensors on the shoes might be difficult for some subjects. Furthermore, the rigidity of fixation and signal features might be affected by the material and shape of the shoes. In contrast, at the waist, a single sensor can easily be attached with minimal discomfort. In our experience, a loose attachment of the sensors to the shoes can cause vibration and displacement of the sensors. Consequently, multiple peaks can occur in the acceleration signal around heel-strike, which may be the reason for some of the differences between the estimates from heel accelerations and Optotrak data, as well as between heel and low-back acceleration signals. In the case of low-back acceleration, the sensor is attached symmetrically on the body in the frontal plane, thus a single sensor can be used to detect steps of both legs. Conversely, for heel accelerometers stride durations are obtained separately for each leg, and step durations are calculated between heel strikes, alternating between legs and thus between sensors. Consequently, a slight misalignment of the sensor from the right to the left heel, or a change in position of the sensor during the gait episode may influence the detection of step durations. Moreover, a different stride cycle template is defined for each of the heel acceleration signals, potentially affecting symmetry when calculating steps. Hence, the evaluation of stride durations instead of step durations might be more consistent when comparing estimates from low-back and heel accelerations. We evaluated this in a post-hoc comparison and found that average absolute differences in stride duration between estimates from low-back accelerations and heel accelerations were on average 19.0 ± 10.6 ms (3.4 ± 1.6 % of average step duration, ICC = 0.98), which is indeed substantially lower (*p* = 0.04) than the comparison in step duration between these methods.

A technical constraint of this study was the failure to continuously track the Optotrak markers, which caused disruption of visibility and precluded the continuous analysis of the signals. As a consequence, 8.9 % of the heel-strike events were not included for the calculation of step durations, and instead, a stride duration was calculated and compared for these cases. Nonetheless, differences in step duration between estimates from low-back accelerations and heel accelerations were not statistically significant different from differences between low-back accelerations and Optotrak. This indicates that despite the reduced number of measured steps, and the comparison of strides instead of steps, the loss of heel-strike events did not have a large impact on accuracy.

In clinical practice, the use of a 3D motion recording system is limited due to technical requirements [1]. Accelerometers on the other hand are small, lightweight, inexpensive, easy to wear, highly transportable, do not require any stationary units, are easy to set up and use, do not require professional operators and their use is not physically constrained [1, 17]. Moreover, in the case of low-back accelerometers, right and left steps can be detected from data collected with a single sensor. However, a main limitation of the present study may be the low sample rate (100 sample/s) of the accelerometers, which may have negatively affected our results. The temporal resolution of the accelerometers was approximately half of the three mean average absolute differences. Consequently, results may improve with higher sample rates. The acceleration pattern (from heel and low-back accelerations) of the intervals around the first heel-strike is different in shape and amplitude from the intervals around the rest of the heel-strikes, especially in the case of heel accelerations. As a result, in the majority of cases the accuracy was lower for the detection of the first heel-strike based on the proposed template-match method. This lower accuracy in detecting the first event has implications for the study of gait initiation, which is relevant in the assessment of short episodes of gait. When excluding the first detected event, differences between systems were lower than the original results including the first event: 17.9 ms (3.2 %, *p* = 0.09) comparing estimates from low-back accelerations and Optotrak, 15.7 ms (2.8 %, *p* = 0.01) between heel accelerations and Optotrak, and 25.9 ms (4.6 %, *p* = 0.71) between low-back and heel accelerations. However, these differences were only significantly different in the comparison between heel accelerometry and Optotrak.

Another limitation of this study was the exclusion of the last two heel-strikes (last step) of the episode of gait. Thiscfinal step is clinically interesting since it challenges the maintenance of balance. However, in this study, the last step corresponded to the positioning phase of the shoes over the footprints, and since some subjects completely stopped before placing their feet over the footprints, the last step could not be considered as part of the continuous gait episode.

Other studies have reported promising results for step detection based on gyroscope signals recorded on the feet [43, 44], with smartphones [45, 46] and in combination with accelerometry signals on the lower back [47]. Moreover, angular velocity signals obtained from gyroscopes at the lower back have been used to differentiate between left and right events [23, 25], to estimate sensor orientation in combination to accelerometry and to obtain step length [26]. In this study, we did not analyse angular velocity signals obtained with gyroscopes, however, this can be the focus of future work.

To our knowledge, there is limited published work related to gait event detection from acceleration signals recorded at the lower back or at the heels over straight gait episodes of short duration. Therefore, it is hard to compare our results to previous literature under the same conditions. An extensive comparison between performances of algorithms in short gait within, as opposed to between sensors, could be a target of future work. Different studies have detected gait events from heel accelerometry based on the detection of peaks in specific regions of filtered signals [18, 35]. However, the validity of the outcomes were not tested in one of the studies [35]. The high accuracy reported in a second study was obtained by performing the validation over gait episodes of longer duration (12 times along a 10 m long path), and thus with more steady gait patterns. In addition, a younger cohort group was assessed (27 ± 2.6 years), which might have resulted in a less variable and less asymmetric gait than in healthy elderly subjects [18]. Other algorithms applied to low-back acceleration signals for step detection have been previously reported [21, 25, 27, 28, 39, 48]. However, the accuracy was not tested in some of them [27, 28], or only average step durations were compared between systems rather than absolute differences between systems [21]. Another study [25] compared the detection of initial contact events from acceleration data recorded on the lower back (L3) with force platforms. Non-absolute differences with high standard deviations (13.4 ± 35 ms) were found for the detection of events. In both previously mentioned studies [21, 25], longer (25 m) episodes of gait were assessed in younger cohorts. The magnitude of the differences obtained in our study is comparable to results found in a previous study [19], which analysed step durations calculated with different methods based on low-back accelerations [20–23, 26], and reported an acceptable accuracy of all of them for clinical use. However, the conditions of the comparative study were different, since the subjects walked a longer distance, while barefoot. Subjects likely walked with different acceleration patterns than with shoes [49] (as in our study), and with a more steady gait pattern. Moreover, in contrast to other methods [19–22, 26], our algorithm performed step detection without false positive and false negatives. Taken together, our results suggest that the proposed algorithm is adequate for assessing short gait episodes in healthy elderly subjects with clinically sufficient accuracy.

Simple and short assessments have potential for inclusion in clinical studies [7, 10, 13], particularly because physical limitations in some patients might be an impediment to perform longer protocols [6, 7]. Moreover, older adults select gait strategies with different spatio-temporal parameters for different distances [8], and since they predominantly perform short bouts of gait in daily-life physical activity [50], the assessment of short episodes of gait may provide clinical information that is different [8] and more relevant than information based on the assessment of long episodes of gait. Additionally, the ability of the elderly to cover indoors distances, which are limited by housing dimensions, is relevant for their safety [51], independence at home and for their daily-life physical activity [52]. Thus, the assessment with BFS of short episodes of gait, extracted from daily-life physical activity or measured in a standardised and supervised setting, may add value in the study of gait quality of the elderly.

The detection of steps is a prerequisite for obtaining spatio-temporal parameters such as cadence, step symmetry, gait variability, anticipatory postural adjustments prior  step initiation, duration of gait initiation, etc., which might have clinical value for the differentiation of stages of neurodegenerative diseases [13, 53–60]. Moreover, step - by - step variability of low-back angular velocity and acceleration might also provide preclinical and progression parameters of such diseases. Thus, if differences in step duration between clinical groups are larger than 4 % on average, as it has been shown in several studies, e.g., in Parkinson’s Disease [56], the algorithm proposed in this study would be useful for the analysis of parameters based on step detection from heel and low-back accelerations.

## Conclusions

The presented study was designed to evaluate the accuracy of a novel algorithm based on acceleration signals recorded at different human locations (lower back and heels) for the detection of step durations over short episodes of gait in healthy elderly subjects. The accuracy was assessed by comparing absolute differences in step duration between three methods: step detection from an optoelectronic 3D motion tracking system, step detection from the application of the algorithm on low-back accelerations, and step detection from the application of the algorithm on heel accelerations.

The proposed algorithm successfully detected all the steps, without false positives and without false negatives. Average absolute differences in step duration within trials and across subjects were calculated for each pair of methods. Differences between methods were on average about 4 %.

From the findings we can conclude that using the proposed algorithm step durations can be estimated with acceptable accuracy by using one of the two methods; placing a single accelerometer device at the lower back of healthy elderly subjects during short episodes of gait, or placing two accelerometers at the heels. This provides opportunities for the extraction of parameters from short episodes of gait, in both clinical settings and possibly in non-supervised environments.
